# Separation of nuclear isomers for cancer therapeutic radionuclides based on nuclear decay after-effects

**DOI:** 10.1038/srep44242

**Published:** 2017-03-13

**Authors:** R. Bhardwaj, A. van der Meer, S. K. Das, M. de Bruin, J. Gascon, H. T. Wolterbeek, A. G. Denkova, P. Serra-Crespo

**Affiliations:** 1 Radiation and Isotopes for Health, Department of Radiation Science and Technology, Faculty of Applied Sciences, Technical University Delft, Mekelweg 15, 2629 JB, Delft, The Netherlands; 2 Catalysis Engineering, Department of Chemical Engineering, Faculty of Applied Sciences, Delft University of Technology, Van der Maasweg 9, 2629 HZ, Delft, The Netherlands

## Abstract

^177^Lu has sprung as a promising radionuclide for targeted therapy. The low soft tissue penetration of its β^−^ emission results in very efficient energy deposition in small-size tumours. Because of this, ^177^Lu is used in the treatment of neuroendocrine tumours and is also clinically approved for prostate cancer therapy. In this work, we report a separation method that achieves the challenging separation of the physically and chemically identical nuclear isomers, ^177m^Lu and ^177^Lu. The separation method combines the nuclear after-effects of the nuclear decay, the use of a very stable chemical complex and a chromatographic separation. Based on this separation concept, a new type of radionuclide generator has been devised, in which the parent and the daughter radionuclides are the same elements. The ^177m^Lu/^177^Lu radionuclide generator provides a new production route for the therapeutic radionuclide ^177^Lu and can bring significant growth in the research and development of ^177^Lu based pharmaceuticals.

Lutetium-177 (^177^Lu) has emerged as a promising radionuclide for targeted therapy. The low energy β^−^ emissions, a half-life of 6.64 days and the emission of low energy and low abundance γ-rays has made ^177^Lu a solid candidate to be the most applied therapeutic radionuclide by 2020^1^. Its low energy β^−^ particles with a tissue penetration of less than 3 mm make it suitable for targeting small primary and metastatic tumours, like prostate, breast, melanoma, lung and pancreatic tumours, for bone palliation therapy and other chronic diseases^2^,^3^,^4^. In addition, the emitted γ rays (208.37 and 112.98 keV) allow simultaneous imaging and quantification of the tumour treatment process *in vivo*^5^. After the success of the pioneering work carried out at Erasmus Medical Centre for treating neuroendocrine tumours with ^177^Lu-labeled peptides, the treatment is now applied at that hospital to more than 400 patients per year^3^,^6^,^7^. Further, the demand of ^177^Lu is expected to grow, since it is now approved for use in prostate cancer treatment and many other treatments are in advanced clinical trial stages^8^,^9^,^10^.

During the production of ^177^Lu by neutron irradiation of lutetium targets, its nuclear isomer ^177m^Lu is formed concomitantly^11^,^12^. Nuclei with the same atomic and mass number but different energy are called nuclear isomers. ^177m^Lu is a high-energy nuclear isomer with a half-life of 160.4 days. 78.6% of ^177m^Lu decays by beta emission to ^177m^Hf and ^177^Hf and 21.4% decays to ^177^Lu, the ground state, via isomeric transition^13^,^14^. Isomeric transition can occur either via internal conversion or γ rays emission (see Fig. 1(a)). Internal conversion is a radiationless decay where the excess of nucleus energy is transferred to an electron in the K- L- or M- shell. This energy transfer leads to an auger electron cascade that results in a highly charged state ultimately provoking bond rupture (see Fig. 1(b))^15^. Nuclear isomers of Te, Co, and Se have been separated in the past using internal conversion^16^,^17^,^18^. However, in these cases bond rupture resulted in chemically separable forms which do not readily undergo exchange, making the separation feasible. In contrast, in case of ^177m^Lu and other isomers, bond rupture leads to chemically and physically alike atoms that cannot be separated with the chemical and physical techniques available. In the present communication, we report a separation method that allows the separation of chemically and physically identical nuclear isomers. The method makes use of the nuclear after-effects caused by the internal conversion to separate the newly formed ground state (^177^Lu) from the metastable state (^177m^Lu).

Our nuclear isomer separation process is based on the combination of three elements (see Fig. 1(b)): (i) a very inert complex with slow association-dissociation kinetics, (ii) the nuclear after-effects of the internal conversion process that breaks the chemical bonds due to the highly charge state created and (iii) a separation method able to set apart the complexed element and the freed one.

This separation method does not only open up the possibility of separating nuclear isomers, but also a novel radionuclide generator for ^177^Lu production can be devised on the basis of the much longer half-life of ^177m^Lu (160.4 days) compared to the 6.7 days half-life of its daughter radionuclide ^177^Lu. Radionuclide generators are in-house production devices that provide a specific radionuclide generated through the decay of a parent radionuclide on demand without the need for access to an isotope producing facility^19^. In this way, the inconvenient dependency for irradiations is eliminated and a constant and continuous availability of the radionuclide of interest is warranted^20^. However, different from the case at hand, all existing radionuclide generators to date are based on the separation of two different elements that can be physically and/or chemically separated^21^. While the use of the metastable ^177m^Lu as the parent radionuclide for ^177^Lu production was proposed by De Vries and Wolterbeek^22^, the realization of a generator has not been demonstrated yet. In order to prove this concept, we have chosen a reversed phase chromatographic system in which ^177m^Lu-DOTA-(Tyr^3^)-octreotate (DOTATATE) complex (with a dissociation constant *k*_*d*_ = 2·10^−8^ s^−1^ at 20 °C) is retained in a tC-18 silica column^23^. The tC-18 silica filler has no affinity toward polar metal ions, and thus the bond ruptured ^177^Lu ions can be eluted off the column using a mobile phase flow, while the ^177m^Lu-DOTATATE complex exhibits a very long retention time with the chosen mobile phase, and remains immobilized on the column during the experiments (shown schematically in Fig. 2(a)). A very similar approach was utilized by Zhernosekov *et al*. to design the ^140^Nd/^140^Pr generator. In this case the parent radionuclide decays via electron capture, a process that leads to an Auger cascade as well. While the parent is retained in a chromatographic column as a complex with DOTATOC, the daughter radionuclide, ^140^Pr, is separated due to the bond rupture effect caused by the Auger cascade that followed the electron capture nuclear transmutation^24^.

## Results

### Continuous elution

Initially, experiments with continuous flow of mobile phase (or continuous elution) were performed at different temperatures and mobile phase fluxes. The initial ^177^Lu/^177m^Lu activity ratio in the ^177m^Lu-complex was measured to be 0.24 ± 0.03. After loading the complex in the column, it was eluted with a continuous mobile phase with a spatial velocity of 0.05 mL/min and 20 °C. Figure 2(b) displays the obtained ^177^Lu/^177m^Lu activity ratio after different elution times. The ^177^Lu/^177m^Lu activity ratio changed in the eluted fractions from the value in equilibrium, app 0.24, to an average ratio of 127 ± 14, accounting for an enrichment in ^177^Lu of more than 500 times. The ratio remains within a small variation up to 60 hours of continuous elution. The gamma spectra before and after separation of the nuclear isomers are displayed in Fig. 2(c,d) respectively. The rather complex decay scheme of the mixture injected in the column, that contains ^177m^Lu, ^177^Lu and ^177m^Hf (Fig. 2c), is in clear contrast with the gamma spectrum of the eluted sample where only the peaks at 113 and 208 keV are observed as the major photo-peaks, from the ^177^Lu decay. The peaks at 249 KeV and 321 KeV are also present in their expected 0.2% relative gamma yields, however the contribution at 321 KeV is enhanced because of the summation effects in the well type germanium detector.

The efficiency of the separation is defined as the ratio of the collected ^177^Lu activity divided by the theoretical activity of ^177^Lu produced from the decay of the parent ^177m^Lu in a specific time (details in supplementary information, equation S1). Specific time being defined as the time during the collection of the elution fraction, which are 6 hours in the case of continuous elution. During these 6 hours a continuous flow of mobile phase is passed through the column, and the total fraction volume collected after 6 hours was used for the measurement. It is important to note that for efficiency calculations, the activity that is not collected in a specific period of time is not considered in the efficiency calculation of the following elution fraction. An average of 64 ± 2% efficiency is obtained for the continuous elution experiments at 20 °C and 0.05 mL/min as shown in Fig. 2(b).

Encouraged by the results on the first ever evidence of ^177m^Lu and ^177^Lu isomer separation, we systematically studied the effect of temperature and elution flux on the activity ratios and efficiency. A range of temperatures from 0 to 30 °C was applied at two different mobile phase fluxes, 0.012 and 0.05 mL/min (all the data can be found in the supplementary information, Figure S1 and Table S1). Figure 3 shows the activity ratio and the efficiency at different temperatures for both fluxes. These data and the corresponding standard deviations are the result of averaging six to eight fractions. The activity ratio is remarkably higher at the lower flux for all the temperatures but 0 °C, reaching an optimum value of 218 ± 10 at 10 °C and 0.012 ml/min. The activity ratio values from 10 to 30 °C show a clear trend for the two fluxes studied, a decrease in their values is observed, reaching a minimum value of 25 ± 3 at 0.05 mL/min and 30 °C. The efficiency exhibits a constant trend for both fluxes in the whole temperature range with slightly higher values for 0.05 mL/min, reaching a maximum value of 65 ± 3% at 10 °C. Only the elutions at 0 °C with a flux of 0.012 mL/min gave a lower efficiency, with a value of 47 ± 4%.

### Accumulation experiments

Accumulation period refers to the total time between elutions during which the flux of mobile phase through the column was stopped. Different accumulation periods up to 5 days were checked at 10, 20 and 30 °C. After a fixed accumulation period, the accumulated activity was eluted with a flux of 0.1 mL/min for 60 minutes. It is optimized after trying different elution fluxes and elution times. The results are summarized in Table S2, Supplementary information.

Further, Fig. 4 displays activity ratio and efficiency as a function of accumulation period. For efficiency calculations, the total accumulation period is used as the ‘specific time’ for the production of ^177^Lu. The activity ratio follows the same trend as in the previous experiments in terms of temperature dependency. Higher activity ratios are observed at low temperatures for different accumulation periods. Moreover, greater ratios than in continuous elution experiments are obtained, reaching a maximum value of 252 ± 12 at 10 °C after 5 days of accumulation, leading to an enrichment factor of around 1000. In contrast, efficiency values are lower than in the continuous elution experiments, decreasing for all the temperatures studied when the accumulation period is extended. No clear trend with temperature is observed. However, in all the cases there is a decrease in efficiency when extending the accumulation period, reaching a minimum above 40%.

## Discussion

The performed method allows for the separation of nuclear isomers. Based on this scheme, we propose a new radionuclide generator for the production of ^177^Lu in which the parent nuclide is the metastable ^177m^Lu. The reversed phase chromatographic column may be operated in a variety of conditions, being possible to modify the temperature, mobile phase flux and operation mode. Despite the fact that the experiments were carried out with low levels of activity, the values were high enough to provide reliable information about the generator performance. In all the analysed fractions, the levels of activity were much higher than the detection limit keeping the measurement error very low.

The importance of the internal conversion process on the bond rupture is clear when our system is compared with the work reported by Severin *et al*. In a very similar chromatographic separation setup, the dissociation of a DOTA-^44m^Sc complex was studied and no bond rupture was observed^25^. ^44m^Sc decays to a large extend through the emission of gamma rays (ICC = 0.1391), while ^177m^Lu decays mainly via internal conversion (ICC = 30.7), therefore having a much greater chance of undergoing bond rupture.

**Figure 1:**
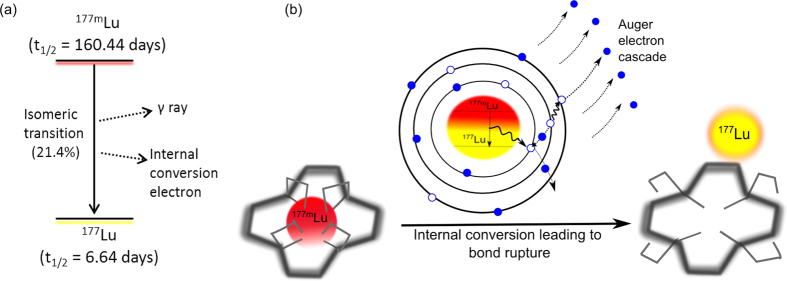
Schematic representation of the decay process. (**a**) Decay scheme of ^177m^Lu to ^177^Lu. (ii) Process of bond rupture. The metastable isomer ^177m^Lu is coordinated to a very stable complex (left side). During the decay via internal conversion the nucleus excess of energy is transferred to an inner electron causing an auger electron cascade (center). After the cascade the atom is in a highly charge state, the chemical bonds are broken and the freed ^177^Lu can be separated (right side).

**Figure 2:**
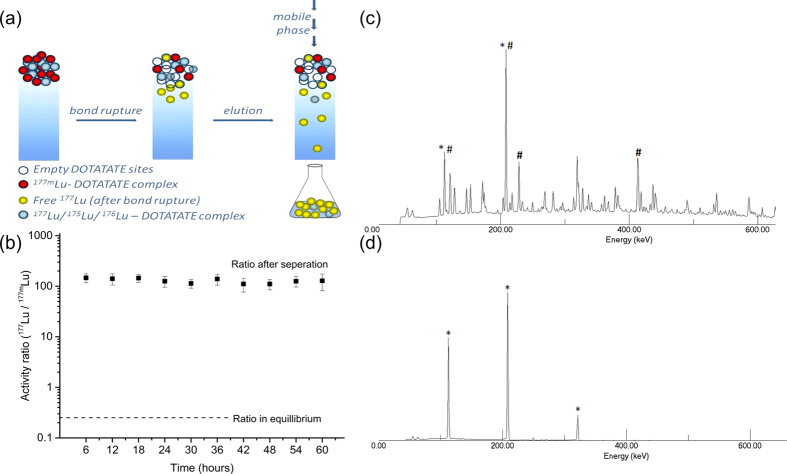
Separation of nuclear isomers ^177^Lu and ^177m^Lu. (**a**) Schematic representation of the experimental setup (**b**) ^177m^Lu/^177^Lu activity ratio at continuous elution with a flux of 13.42 mm/min and a temperature of 20 °C. (**c**) γ ray spectra of the mixture injected in the column with photo-peaks having contribution from both ^177^Lu(*) and ^177m^Lu(#). (**d**) γ ray spectra of eluted fraction after separation with major photo-peaks from ^177^Lu(*), less than 0.5% contribution from ^177m^Lu.

The generator operates in a reliable and constant fashion, as it can be observed in Fig. 1(b). After 60 hours of continuous operation the activity ratio values differed only a 1.1% from the average. This reliability is very important due to the long half-life of the parent nuclide, what will assure a proper and constant functioning during the long operative life of the generator. The flow rate applied in the elutions is limited by the retention of the Lu-DOTATATE complex. Higher fluxes than 0.1 mL/min lead to a displacement of the complex. The elution rates were selected in order to minimize this effect as much as possible.

**Figure 3:**
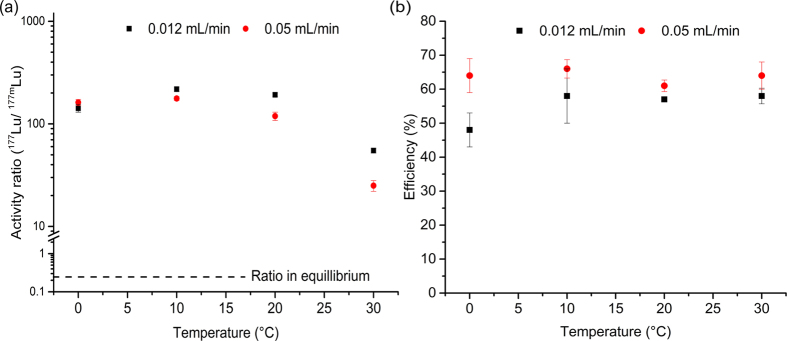
Effect of temperature and flow rate on efficiency. (**a**) Effect of temperature on the^177^Lu/^177m^Lu activity ratio at different flow rates (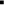 0.012 mL/min and 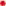 0.05 mL/min). (**b**) Effect of temperature on the efficiency of separation at different flow rate (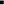 0.012 mL/min and 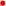 0.05 mL/min).

**Figure 4:**
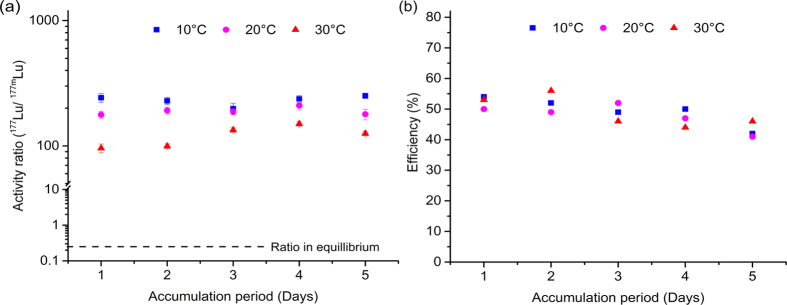
Effect of ^177^Lu activity accumulation on ratio and efficiency. Accumulation period is the total time between elutions while there is not mobile phase flux. (**a**)^177^Lu/^177m^Lu activity ratio obtained after accumulation period at different temperatures (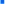
10 °C, 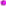
20 °C, 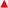
30 °C). (**b**) Efficiency of separation v/s the accumulation time at different temperatures (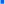
10 °C, 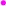
20 °C, 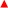
30 °C).

Temperature shows an important effect on the generator separation performance. It is expected because the association-dissociation kinetics of the Lu-DOTATATE complex are highly influenced by temperature. Dissociation kinetics of Lu-DOTATATE complex were reported by Van der Meer *et al*. using a similar system and an order of magnitude difference was calculated when the temperature was increased from 20 to 37 °C^23^. A higher dissociation rate turns into a higher concentration of dissociated ^177m^Lu in the mobile phase decreasing the value of the activity ratio and the quality of the elution. Oppositely, the rate of production of ^177^Lu by internal conversion is independent of temperature, and is only time dependent. The change in the activity ratio at different temperature is comparable in both modes of operation, continuous elution and accumulation (see Figs 3(b) and 4(b)). The optimal temperature is found to be 10 °C, where maximum values of the activity ratio are achieved for both cases. The experiments at 0 °C showed results in contradiction with the above explanation of using low temperatures. This can be explained by the fact that at 0 °C there might be some other effects that can alter the operation of the generator. Temperature of 0 °C is close to the freezing point of the mobile phase and mass transfer of the freed ^177^Lu may be hindered, limiting the amount of eluted ^177^Lu and decreasing the values of the activity ratio and the efficiency.

In clear contrast, temperature does not show any effect on the efficiency of the collected ^177^Lu in any of the operation modes (see Figs 3(a) and 4(a)). The efficiencies are not close to the ideal value of 100%. It can be because of (*i*) some loss of ^177^Lu ions by adsorption in different parts of the column (*ii*) the uncertainty from the internal conversion process since the efficiency of it, to the best of our knowledge, is unknown. The internal conversion process leads to a complex situation that eventually leads in some cases to bond rupture. Before an accurate value of efficiency of the generator can be given, more needs to be known about the internal conversion process and its effectiveness in the rupture of chemical bonds.

The effect of the elution rate on the activity ratio and efficiency during the continuous elution experiments can be explained on the basis of observations at different conditions. If the column is eluted with a higher flux than 0.1 mL/min a displacement of the ^177m^Lu-DOTATATE complex is observed and the activity ratio measured are much worse with greater amounts of ^177m^Lu. The same may be occurring to some extend when the flux of 0.05 mL/min is compared with 0.012 mL/min, and small amounts of the complex might elute through the column, decreasing the activity ratio (see Fig. 3(b)). Moreover, the low flow might not be enough to provide good mass transfer to the freed ^177^Lu ions and some of them may re-associate back to the ligand, decreasing in this way slightly the efficiency of the elution (see Fig. 3(a)).

The effect of the re-association may also explain the results observed in the accumulation experiments. A remarkable decrease in the efficiency is observed when the accumulation time is extended (Fig. 4(a)). In long periods of time the chances of re-association of freed ^177^Lu back to the free DOTATATE molecules increases, which decreases the concentration of freed ^177^Lu in the elutions, and therefore the efficiency. However, in the case of the activity ratio it has a positive effect, as re-association will decrease the amount of ^177m^Lu ions thereby incrementing the activity ratio values obtained (Fig. 4(b)) in comparison with the continuous elution. In continuous elution, the collected ^177m^Lu is produced by the exclusive contribution of complex dissociation and the eluted ^177^Lu is due to the combination of complex dissociation and bond rupture. In the case of accumulation, re-association takes place decreasing in the same proportion the concentration of both isomers in the mobile phase. Since the contribution by bond rupture is not altered during the accumulation, the activity ratio value increases due to this phenomenon.

The ^177m^Lu/^177^Lu generator could complement the present ^177^Lu production routes. In the current situation, two different production routes are established: the indirect and direct routes^11^,^12^,^26^. In the indirect route, ^177^Lu is produced as the decay product of the short-lived ^177^Yb, which is produced by neutron capture of enriched ^176^Yb^27^. Despite the fact that no-carrier added ^177^Lu is produced in this process, the high cost of enriched ^176^Yb and the radiochemical separation of ^177^Lu from the ^176^Yb target are limiting its application^28^,^29^,^30^. The direct route produces ^177^Lu by neutron capture of enriched ^176^Lu with clinically required specific activity at a lower cost^31^,^32^. However, as previously mentioned, during the neutron irradiation the long-lived metastable ^177m^Lu (T_1/2_ = 160.44days) is co-produced, causing a problem in the waste management of medical centres^7^,^33^. On top of these issues, both routes depend on the constant availability of nuclear reactors since weekly irradiations are needed for the production of ^177^Lu^34^.

The direct route of producing ^177^Lu provides hospitals with a maximum relative activity of ^177m^Lu of 0.01–0.02% at the end of bombardment^11^. The maximum activity ratio obtained in our experiments is about 250, when expressed in the same fashion accounts for a value of about 0.4%. The aim of our system is to prove that both isomers can be separated based on the internal conversion process. A clinical ^177m^Lu/^177^Lu generator would need much higher levels of activity and a high resistance to radiation damage that would allow a consistent performance along the life-time of the device. In order to go beyond the proof of concept, the use of more stable chemical complexes will be combined with higher activities. This will allow the creation of a generator with a more flexible and robust performance that may produce non-carrier added ^177^Lu and will permit a realistic evaluation of a potential generator for clinical use. Such a generator would combine the benefits of the direct and indirect routes, providing a product with higher quality that would eliminate the problems associated with the waste management due to the presence of ^177m^Lu. Further, the half-life of the parent radionuclide will assure a stable production of ^177^Lu for months and therefore the generator would terminate the need of weekly irradiation, and the continuous dependency on nuclear reactors to produce ^177^Lu. With this generator more possibilities will be open for the use of ^177^Lu in more hospitals and research centres. In contrast, the production of enough ^177m^Lu to supply the generators with enough activity will need to be thoroughly examined in order to evaluate the feasibility of the clinical application of the ^177m^Lu/^177^Lu generator.

Summarizing, the separation of the nuclear isomers ^177m^Lu/^177^Lu has been achieved by taking advantage of bond rupture upon decay in a method that may be applied to other mixtures of isomers. In order to achieve the separation, a complex with very high stability needs to be formed with the metastable isomer. The nuclear after-effects of the internal conversion decay process, in which ^177m^Lu is transmuted to ^177^Lu, lead to chemical bonds breakage and the ^177^Lu ions becoming free. By means of retaining the complex ^177m^Lu-DOTATATE in an apolar chromatographic column, the freed ^177^Lu can be separated by fluxing the column with a mobile phase with the proper polarity. In this way, a new type of radionuclide generator is conceived, in which both the parent and the daughter nuclides are the same element. The generator will open new possibilities for the production and availability of the therapeutic radionuclide ^177^Lu and can bring significant growth in the research and development of ^177^Lu based pharmaceuticals.

## Methods and Materials

### Materials

The ^177m^Lu activity source was provided by IDB Holland. It was approximately 1 mM LuCl_3_in 1 M HCl solution with a specific activity of 7.2 MBq/g of LuCl_3_. In preliminary experiments, LuCl_3_ was neutron activated in the HOR reactor at Delft, however the long irradiation times needed made the supply by IDB Holland preferred. DOTATATE (Biosynthema) was provided by the Erasmus medical center, Rotterdam. Reversed phase material, tC-18 silica was purchased in the form of ready to use sep-pak cartridges (Sep-Pak Plus tC18, usable for pH 2–8), from Waters.

### Synthesis of ^177m^Lu-DOTA-(Tyr^3^)-octreotate complex

The ^177m^Lu solution was adjusted to pH 4 using 1 M NaOH solution, 20 μL of 1 M NaAc-HAc buffer was also added to keep the pH around 4 during the reaction. Lu-DOTA-(Tyr^3^)-octreotate, also referred as Lu-DOTATATE, was synthesized using 0.150 μmoles Lu (150 μL of 1 mM LuCl_3_ solution, app. 1 MBq^177m^Lu) and 0.278 μmoles DOTATATE leading to a total reaction mixture volume about 1 ml. The reaction mixture was then incubated at 80 °C for 1 hour. The completion of the reaction was checked using instant thin layer chromatography with 1:1 acetonitrile: water as the mobile phase, and silica as the stationary phase. The reaction conditions resulted in >99% complexation yield.

### Experimental setup description

The experimental set up consists of an HPLC-system consisting of a pump (Shimadzu LC-10Ai), PEEK tubing and a fraction collector for 20 ml vials. The pump was connected to a column made of peek (ID 3 mm × 47 mm). The column was manually filled with tC-18 reversed phase silica (waters). A slurry of tC-18 silica in MeOH was added from one end of the column and the other end was connected with a vacuum pipe. The empty column has a volume of 0.335 mL, after filling the column with silica the void volume is experimentally calculated to be 0.175 mL (details in supplementary info S7). The column was then equilibrated with the mobile phase for overnight before injecting the complex. The mobile phase and column were both temperature controlled to the desired temperature by a thermostatic circulation water bath (Colora WK4) and a column water jacket (Alltech). 0, 10, 20, and 30 °C were the studied temperatures.

### Mobile phase composition

The mobile phase consists of 5% methanol, 150 mM NaCl solution (ionic strength of 0.148 M), 10 mM NaAc- HAc buffer (pH-4.3). Mobile phase flux of 0.012 and 0.05 mL/min were used during continuous elution, and 0.1 mL/min is used during accumulation experiments. The whole experimental setup was equilibrated for at least two hours with the mobile phase prior to loading of the complex.

### Loading of the complex

The complex was loaded on the manually filled tC-18 column using a Rheodyne injector, with a mobile phase flow of 0.1 mL/min. Prior to injection, a 2 μl aliquot was kept aside and measured to know the exact activity loaded on the column. During the first 30 minutes the flow was set to 0.1 mL/min to remove free metal and impurities or side-products. The eluted fraction was then used to measure the amount of activity lost in loading, as impurities/side products. After the first fraction of 1 hour, the flow rate through the column was adjusted to the desired flow rate at the desired temperature. The same procedure was repeated with 3 different columns and 3 different Lu-DOTATATE complexes with the initial activity around 1.45 ± 0.04 MBq, 0.65 ± 0.02 MBq, 0.98 ± 0.03 MBq. In all the cases the total initial activity retained on the column is about 98% of the initial activity. Once loaded, a column is used upto 3 months for doing the measurements. After a maximum period of 3 months, the column is flushed with pure methanol to remove all the loaded activity.

### γ ray spectroscopy analysis

All the fractions were measured on well-type HPGe detector. The efficiency calibration for different peaks was performed using a known activity of Lu-177 source supplied by IDB Holland. The fraction volumes up to 18 ml were collected during the experiments, however all the measurement were performed with a fixed 0.4 ml aliquot for a time period of 3 hours. The gamma ray spectra were analyzed using an in-house software^35^ to calculate the activity in (Bq/g) of each fraction. The activity concentration obtained in Bq/g was then multiplied with the total mass of the fraction to know the absolute activity coming out in each fraction. To minimize the error, all the vials were weighed before and after the fraction collection.

### Continuous elution

In the continuous elution mode two flow rates were studied, 0.012, 0.05 mL/min at 10, 20, and 30 °C. For each flow rate and temperature six to eight fractions were collected for 6 hour each. Each fraction was measured on the above mentioned well type germanium detector. The individual results and calculations can be found in supplementary information in sections S2 and S3.

### Accumulation followed by elution

For accumulation experiments the flow of mobile phase was stopped in the column for 1, 2, 3, 4, 5 days at 10, 20, and 30 °C respectively. For flushing the accumulated activity the flow rate of 0.1 mL/min was used and fraction collected in the first 60 minutes was used to measure the efficiency, ^177^Lu/^177m^Lu activity ratios. In accumulation experiments, the error bars in activity ratio plots define the instrumental error in the measurement while for efficiency measurements the errors are less than 1%, so they are not shown in the graphs. Detailed results and explanations are given in supplementary info S5.

## Additional Information

**How to cite this article:** Bhardwaj, R. *et al*. Separation of nuclear isomers for cancer therapeutic radionuclides based on nuclear decay after-effects. *Sci. Rep.* **7**, 44242; doi: 10.1038/srep44242 (2017).

**Publisher's note:** Springer Nature remains neutral with regard to jurisdictional claims in published maps and institutional affiliations.

## Supplementary Material

Supplementary Information
