# Supplementary material for: Separation of nuclear isomers for cancer therapeutic radionuclides based on nuclear decay after-effects
Source: Sci Rep. 2017 Mar 13;7:44242. doi: 10.1038/srep44242 (PMC5347157; doi:10.1038/srep44242)
Supplement: Supplementary Information [file srep44242-s1.pdf]

Supplementary information

Separation of nuclear isomers for cancer therapeutic radionuclides based on nuclear decay after-effects

R. Bhardwaj[1,2], A. van der Meer[1], S.K. Das[1], M. de Bruin[1], J. Gascon[2], H. T. Wolterbeek[1], A. G. Denkova[1]and P. Serra-Crespo[1,*]

[1]Radiation and Isotopes for Health, Radiation Science and Technology, Faculty of Applied Sciences, Technical University Delft, Mekelweg 15, 2629 JB, Delft, The Netherlands

[2]Catalysis Engineering, Chemical Engineering, Faculty of Applied Sciences, Delft University of Technology, Van der Maasweg 9, 2629 HZ, Delft, The Netherlands

S1: Efficiency of the ^{177m}Lu/ ^{177}Lu generator

The efficiency of ^{177m}Lu/ ^{177}Lu generator is defined as the ratio of the collected ^{177}Lu activity divided by the theoretically produced ^{177}Lu activity.

Theoretical production in time t is estimated by using the equation;

$$efficiency(\%) = \frac{A_g^t\ (collected)}{A_m^0 \cdot \left(\frac{\lambda_g}{\lambda_g - \lambda_m}\right) \cdot \left[exp^{-\lambda_m.t} - \ exp^{-\lambda_g.t}\right] \cdot B.R \cdot P.I.C.} \cdot 100$$

where A_m^0 = Initial activity of ^{177m}Lu before elution,

λ_g, λ_m = decay constants of ^{177}Lu, ^{177m}Lu respectively,

A_g^t = collected activity of ^{177}Lu at time t,

B.R = branching ratio for ^{177m}Lu to ^{177}Lu decay, 21.4% [1],

P.I.C = probability of internal conversion, $\frac{\alpha}{1+\alpha}$, 96.8%

where α is known as the internal conversion coefficient, and is defined as;

$$\alpha = \frac{number\ of\ de-excitations\ by\ the\ release\ of\ conversion\ electrons}{number\ of\ de-excitations\ via\ gamma\ ray\ emission}$$

Hence, we define the probability of the decay following the internal conversion path as, P.I.C = $\frac{\alpha}{1+\alpha}$. The 116KeV transition involved in the decay of ^{177m}Lu to ^{177}Lu has a theoretical internal conversion coefficient value, α_{th} = 30.7 [2, 3]. Thus the P.I.C value is calculated to be 96.8%.

S2: Efficiency plots, while having a continuous flow of mobile phase:

As mentioned before, for each flow rate and temperature six to ten measurements were done and their average along with the standard deviation are plotted in figure S2.1

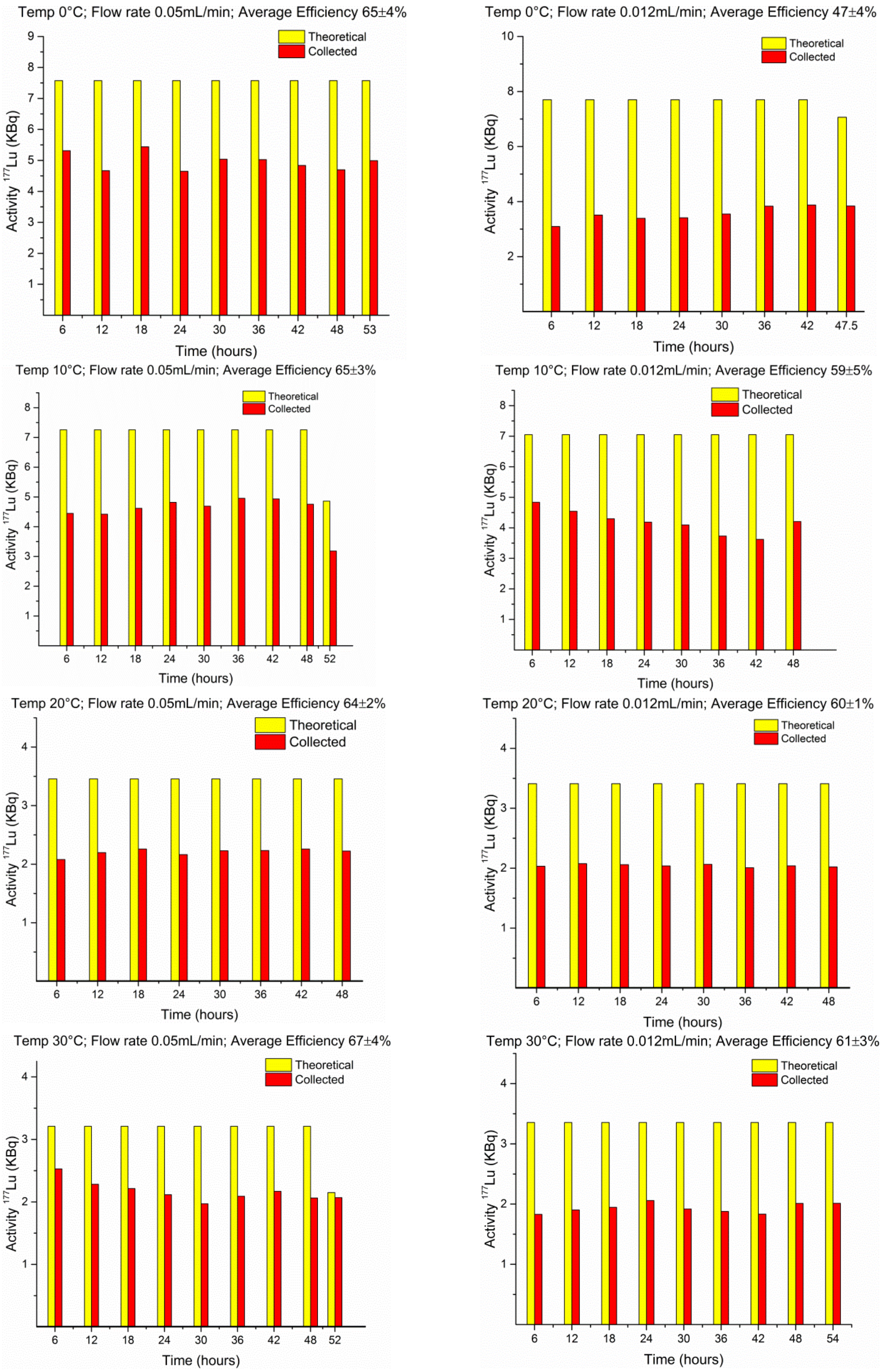

Figure.S1: Efficiency of accumulation at different temperature and flow rates

S3: Ratios obtained in different fractions

Table S1: Ratios obtained at different temperatures, and flow rates

Fraction number	Activity Ratio ($^{177}Lu/ ^{177m}Lu$)							
	at 0°C		at 10°C		at 20°C		at 30°C	
	0.05 mL/min	0.012 mL/min	0.05 mL/min	0.012 mL/min	0.05 mL/min	0.012 mL/min	0.05 mL/min	0.012 mL/min
**1**	160	134	174	213	147	198	21	56
**2**	147	131	184	211	141	206	23	51
**3**	132	135	166	216	145	181	24	55
**4**	168	136	168	209	126	198	30	52
**5**	140	146	183	223	139	190	26	58
**6**	171	158	187	238	106	185	23	59
**7**	194	161	179		111	190	25	58
**8**	160				111		26	51
**9**	178				126			
**10**	170				128			
**Avg±STD**	162±12	143±12	177±8	218±11	126±14	192±8	25±3	55±3

S4: Optimisation of elution flux and elution times for accumulation experiments

To optimize the elution flow rate and elution times, we did different accumulations and then different flow rates are used to elute the accumulated activity. The results obtained are summarized below:

Table S2: Optimisation of elution time, elution flux for accumulation experiments

Elution Flux (mL/min)	Elution Time (min)	Elution efficiency	Remark
0.012	120	About 12%	-
0.1	60	> 60%	-
0.5	60	> 100%	More than 100% efficiencies and very poor $^{177}Lu/^{177m}Lu$ ratios (less than 1), indicates the displacement of complex from the column.

Further, to minimize the volume of eluted activity and to keep the dilution of eluted activity as low as possible. We studied elution profile of Lu-177 after accumulation for an hour while taking the fractions every 5 mins. The result are shown in the plot Figure S4.2. As seen from the plot, a trailing behaviour in the elution of Lu-177 is observed. After elution for about 60 minutes, 60% of the accumulated activity could be removed. Therefore we decided to do the elution of accumulated activity at 0.1 mL/min for 60 minutes.

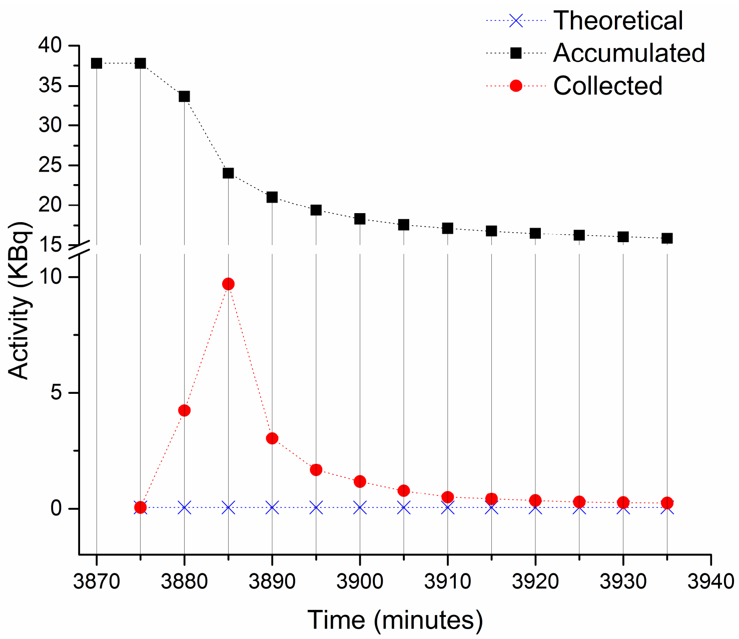

Figure S2. Optimization of elution time while eluting the accumulated activity at 0.1 mL/min.

S5. Detailed results from accumulation experiments.

For accumulation experiments, we were mainly interested in knowing if the separation of the isomers is possible for different accumulation periods. As shown in the Figure 4 of main text, the activity ratios and efficiencies follow almost a constant behaviour, with no substantial change at a particular temp. There was no big deviation from separation, and even under no mobile phase flow for time period upto 5 days the system was capable to separate the two isomers.

Therefore we didn't took many reading for a same experimental point. We did repeat some of these observations twice which gave quite consistent data, the results are shown in the table below

Table S3: ^{177}Lu/^{177m}Lu ratio and efficiency obtained for different accumulation periods at 10, 20 and 30°C

Accumulation time	Fraction number	10°C		20°C		30°C	
		Ratio	Efficiency	Ratio	Efficiency	Ratio	Efficiency
1 day	1	242	56	177	11	96	60
	2	200	50	-	-	67	53
	Average	221	53	-	-	82	56
	STDEV	29	4	-	-	21	5
2 day	1	228	52	191	49	99	59
	2	-	-	240	47	83	56
	Average	-	-	216	48	91	57
	STDEV	-	-	35	1	11	2
3 day	1	198	51	126	43	134	49
	2	-	-	188	53	-	-
	Average	-	-	156	48	-	-
	STDEV	-	-	44	7	-	-
4 day	1	237	15	210	50	150	46
		-	-	-	-	190	44
	Average	-	-	-	-	170	45
	STDEV	-	-	-	-	28	2
5 day	1	251	41	179	45	126	46

S6. Summary of the continuous and accumualtion experiments;

For a better understanding of the data presented in Figure 3 and Figure 4, the results are summarized in table 2 and 3 below:

For continuous flow of mobile phase

Table S4: Summary of the $^{177}Lu/^{177m}Lu$ activity ratios and efficiency obtained under continuous elution mode at 0, 10, 20, and 30°C

Temperature/ °C	$^{177}Lu/^{177m}Lu$ activity ratio		Efficiency (%)	
	0.012 mL/ min	0.05 mL/ min	0.012 mL/ min	0.05 mL/ min
0	142 ± 12	162 ± 11	47 ± 4	65 ± 4
10	218 ± 11	177 ± 8	60 ± 5	65 ± 3
20	192 ± 8	119 ± 11	60 ± 1	64 ± 2
30	55 ± 3	25 ± 3	61 ± 3	67 ± 4

For accumulation and elution experiments

Table S5: Summary of the $^{177}Lu/^{177m}Lu$ activity ratios and efficiency obtained under accumulation elution mode at 10, 20, and 30°C for an accumulation period of 1, 2, 3, 4, 5 days.

Accumulation time/ day	$^{177}Lu/^{177m}Lu$ activity ratio			Efficiency (%)		
	10°C	20°C	30°C	10°C	20°C	30°C
1	242 ± 20	177 ± 11	96 ± 8	56	50	53
2	229 ± 15	191 ± 12	99 ± 4	52	49	56
3	198 ± 20	125 ± 3	134 ± 6	50	54	47
4	237 ± 15	210 ± 16	150 ± 6	51	47	44
5	251 ± 12	178 ± 17	126 ± 5	43	44	48

S7. Determination of void volume of the column and linear velocities

After filling the column with stationary phase, tC-18 silica, we determined the void volume of the column in order to have an idea about the linear velocities of the mobile phase through the column. The experiment set up involved for determining the void volume of the column is shown in Figure 3. A peek column with dimensions diameter 3 mm and length 47 mm is filled with tC-18 silica. The column is connected with an injector, UV cell and a fraction collector using tubings of known volume (a,b,c).

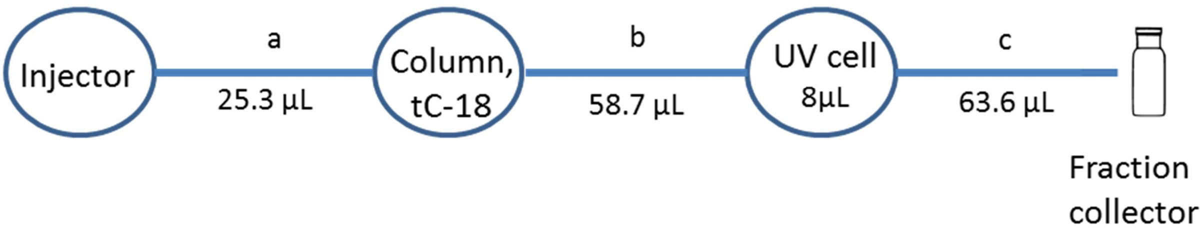

Figure S3. Experimental setup for void volume determination.

1 M $NaNO_3$ is then used as a marker to determine the void volume and it is injected through the injector at two different flow rates 0.3 mL/min and 1.0 mL/min. Once a signal is observed in UV detector, the mobile phase flow through the column is stopped. The results obtained are shown in table 6;

Table S6: Results for void volume determination

Flow (mL/min)	Marker	Time (sec)	n	SD	Volume (µL) Injector + Column – UV cell (a+b+c)	Volume (µL) Injector + Column (a+b+c) - c	Volume of column (a+b) - a
0.30	$NaNO_3$	51.97	5	0.86	257.84	201.14	175.84

The observed void volume is about 50% of the column volume. Using 0.175 mL as the void volume the linear velocities through the column can be calculated as;
For 0.1 mL/min - 26 mm/min, for 0.05 mL/min – 13.42 mm/min, for 0.012 mL/min – 3.22 mm/min.

[1] F.G. Kondev, Nuclear data sheets for A = 177, Nuclear Data Sheets, 98 (2003) 801- 1095.
[2] BrIcc program package version 2.2b, (2009).
[3] T. Kibédi, T.W. Burrows, M.B. Trzhaskovskaya, P.M. Davidson, C.W. Nestor Jr, Evaluation of theoretical conversion coefficients using BrIcc, Nuclear Instruments and Methods in Physics Research Section A: Accelerators, Spectrometers, Detectors and Associated Equipment, 589 (2008) 202-229.
